# CLK1 is a potential tumor suppressor for NSCLC by regulating cell proliferation and immune infiltration

**DOI:** 10.3389/fcell.2026.1758135

**Published:** 2026-02-09

**Authors:** Rui Ma, Xiaoyan Zhang, Yunlong Wang, Jia Ma

**Affiliations:** 1 College of Life Science and Technology, Huazhong Agricultural University, Wuhan, China; 2 College of Biomedicine and Health, Huazhong Agricultural University, Wuhan, China; 3 State Key Laboratory of Biocatalysis and Enzyme Engineering, School of Life Sciences, Hubei University, Wuhan, China; 4 Department of Radiation Oncology, Hubei Cancer Hospital, Tongji Medical College, Huazhong University of Science and Technology, Wuhan, China

**Keywords:** bioinformatics, CLK1, immune infiltration, non-small cell lung cancer, tumor suppressor factors

## Abstract

**Background:**

Lung cancer is the malignancy with the highest global incidence and mortality. Non-small cell lung cancer (NSCLC) accounts for approximately 85% of cases and is characterized by complex drug resistance and poor prognosis. While CLK1 has been implicated in Alzheimer’s disease, pancreatic cancer proliferation, and chemotherapy resistance in lymphoma, its role in NSCLC, particularly in the context of tumor immune infiltration, remains unexplored.

**Methods:**

CLK1 expression and prognostic significance were analyzed across cancers and in LUAD using bioinformatics platforms (GEPIA, UALCAN). Functional enrichment analyses (GSEA, KEGG, GO) elucidated associated pathways and immune correlations. Drug sensitivity screening (GDSC, CTRP, CellMiner) identified potential compounds targeting CLK1-high tumors. Experimental validation was performed using clinical samples from NSCLC patients (n = 12) and *in vitro* assays with A549 and H1299 cell lines to assess CLK1 expression and its effect on proliferation.

**Results:**

Contrary to its oncogenic role in other cancers, CLK1 acts as a tumor suppressor in NSCLC. High CLK1 expression correlated with prolonged survival, suppressed cell cycle and metabolism pathways, and enhanced anti-tumor immunity—particularly CD4^+^ T cell infiltration. Clinically, high CLK1 was associated with increased tumor mutational burden and greater sensitivity to chemotherapy. Consistent with this, CLK1 was downregulated in NSCLC patient tissues of NSCLC, and its overexpression directly inhibits cancer cell proliferation *in vitro*.

**Conclusion:**

Our findings demonstrate that CLK1 functions as a tumor suppressor gene in NSCLC, inhibiting proliferation and promoting immune infiltration. It also correlates positively with sensitivity to multiple chemotherapeutic agents. Thus, CLK1 may serve as a novel prognostic biomarker and a potential target for combination therapy in NSCLC.

## Introduction

According to 2024 global cancer statistics (primarily based on 2022 data), lung cancer remains the leading cause of cancer-related morbidity worldwide, exhibiting accounting for 12.4% of new cases and 18.7% of cancer deaths (Bray et al., 2024). Non-small cell lung cancer (NSCLC) constitutes approximately 85% of all lung cancer cases. While early screening has reduced mortality by 20%, and advances in immunotherapy and targeted therapy have significantly improved outcomes, the overall prognosis remains poor. Recent data indicate that the 5-year survival rate for lung cancer patients is still below 20%, with a high rate of recurrence (Alduais et al., 2023). Therefore, further investigation into the underlying molecular mechanisms of NSCLC is imperative.

Over the past decade, treatment for NSCLC has evolved from conventional surgery, radiotherapy, and chemotherapy toward precision modalities, including targeted therapy, immunotherapy, and antibody-drug conjugates (ADCs) (Le Pechoux et al., 2025). Despite these advances, drug resistance—particularly to immunotherapy—remains a major clinical challenge. Prolonged Immunotherapy can lead to immune evasion, significantly limiting its long-term efficacy (Le Pechoux et al., 2025). This underscores the need to identify novel immune-related therapeutic targets in NSCLC.

The CLKs (Cdc2-like kinases) family, part of the CMGC kinase group, includes four highly homologous members in mammals: CLK1, CLK2, CLK3, and CLK4 (ElHady et al., 2023). These dual-specificity kinases are characterized by a conserved LAMMER motif, and play diverse roles in cancer (Duncan et al., 1995). For example, CLK2 inhibition has shown antitumor effects in breast cancer models (Zhu et al., 2018), while the CLK1-SRSF5 axis promotes proliferation in pancreatic cancer by inducing Cyclin L2 splicing (Chen et al., 2021). Despite these insights, the function of CLK1 in NSCLC—particularly its influence on the tumor immune microenvironment—remains largely unexplored.

Based on this gap in knowledge, we hypothesized that CLK1 may play a distinct role in NSCLC progression and immune regulation. To test this, we designed the present study to address the following research questions: (1) What is the prognostic significance of CLK1 expression in NSCLC? (2) How does CLK1 influence biological pathways and immune cell infiltration within the tumor microenvironment? (3) Can CLK1 serve as a predictive biomarker for therapy response?

We employed bioinformatics and experimental approaches to investigate these questions. Gene Set Enrichment Analysis (GSEA) was used to identify CLK1-associated signaling pathways. Immune infiltration analysis was conducted to evaluate its correlation with immune cell profiles and patient prognosis. Our findings suggest that CLK1 may represent a novel prognostic marker and a potential immunomodulatory target in NSCLC.

## Materials and methods

### Cell culture

The human NSCLC cell lines A549 and H1299 were obtained from Procell Life Science & Technology Co., Ltd. (Wuhan, China). All cell lines were cultured in Dulbecco’s modified Eagle’s medium (DMEM; Gibco) supplemented with 10% fetal bovine serum (FBS; Gibco) and 1% penicillin/streptomycin (Biosharp) at 37 °C a humidified incubator with 5% CO_2_. All cell lines were routinely confirmed to be free of *mycoplasma* contamination. For stable overexpression of CLK1 (pHAGE-CLK1), the full-length human CLK1 cDNA was cloned into a lentiviral expression vector pHAGE-N-HA-C-FLAG. Lentiviral particles were produced by co-transfecting HEK293T cells with pHAGE-CLK1 (2.4 μg) and the packaging plasmids psPAX2 (1.2 μg) and pMD2. G (1.2 μg) using Neofect™ DNA transfection reagent (Neofect™) 5 μL. The viral supernatant was collected 48–72 h post-transfection. A549 and H1299 cells were infected with the viral supernatant in the presence of 8 μg/mL polybrene (Sigma-Aldrich). Stable polyclonal populations were selected and maintained using 2 μg/mL puromycin (Solarbio).

### Cell growth and cell viability assay

For cell growth analysis, control and CLK1-overexpressing cells were seeded in 6-well plates at a density of 3 × 10^4^ cells per well. Cells were harvested by trypsinization and counted manually using a cell counter at 24-, 48-, and 72-h post-seeding. Cell viability was assessed using the Cell Counting Kit-8 (CCK-8, Biosharp, BS350A). Cells were seeded in 96-well plates at a density of 3 × 10^3^ cells per well. At the indicated time points, 10 µL of CCK-8 reagent was added to each well and incubated for 1 h at 37 °C. Absorbance was measured at 450 nm using a microplate reader at 24-, 48-, and 72-h post-seeding. All cell counting and CCK-8 assays were performed with at least three independent biological replicates, each containing three technical repeats.

### Immunoblotting analysis

According to our previous preparation method (Ma et al., 2019). Protein lysates from human tissue samples or cultured cells were extracted using RIPA lysis buffer (Biosharp, BL504A) containing protease inhibitors (cocktail and PMSF). Protein concentration was determined using a BCA assay kit (Beyotime). Equal amounts of protein were separated by 10% SDS-PAGE gel and transferred onto PVDF membrane (Absin). Membranes were blocked with 5% non-fat milk for 1 h at room temperature and then incubated overnight at 4 °C with primary antibodies: rabbit anti-CLK1 (1:500; Proteintech, 20439-1-AP); and rabbit anti-β-actin (1:5000; ABclonal, AC038). After washing, membranes were incubated with an HRP-conjugated goat anti-rabbit secondary antibody (1:5000; ABclonal) for 2 h at room temperature. Protein bands were visualized using an enhanced chemiluminescence (ECL) detection system (Biosharp).

### Datasets and data preprocessing

RNA-seq expression profiles (FPKM-UQ), somatic mutation data, and clinical information for Lung Adenocarcinoma (LUAD) were retrieved from The Cancer Genome Atlas (TCGA) using the TCGAbiolinks R package (Colaprico et al., 2016). Normal tissue samples and tumor tissue samples with incomplete clinical information were excluded, resulting in a final cohort of 455 LUAD patients with complete data. Patients were stratified into CLK1-high and CLK1-low expression groups based on the median CLK1 expression value.

Pan-cancer analysis of CLK1 expression was performed using the GEPIA (GEPIA) database. Protein expression data for CLK1 in LUAD were obtained from the UALCAN (https://ualcan.path.uab.edu/). Immunohistochemistry images were sourced from the Human Protein Atlas (HPA) (The Human Protein Atlas) database.

### GSEA and functional pathway analysis

Differentially expressed genes (DEGs) between the CLK1-high and CLK1-low groups were identified using the DESeq2 R package (version 1.46.0). DEGs were defined with an adjusted p-value (padj) < 0.05 and an absolute fold change (|FC|)>1.5. GSEA was performed using the ClusterProfiler R package (version 4.4.2) with the ‘c2. cp.kegg.v7.2. symbols’ gene set collection. CLK1 expression levels were used as a phenotypic label. Enrichment results with a normalized enrichment score (|NES|)>1, a false discovery rate (FDR) < 0.25, and a nominal p-value<0.05 were considered statistically significant. Kyoto Encyclopedia of Genes and Genomes (KEGG) and Gene Ontology (GO) analyses of the significantly DEGs were conducted using the ClusterProfiler R package (version 4.4.2).

### Survival analysis

The prognostic value of CLK1 expression was assessed using Kaplan-Meier (K-M) analysis. Patients were dichotomized into high and low groups based on the median CLK1 expression. Overall survival (OS) and lung cancer-specific survival (LCSS) were used as endpoints. Log-rank tests were used to compare survival curves. External validation of CLK1’s prognostic role in LUAD and Lung Squamous Cell Carcinoma (LUSC) was evaluated using the KM plotter online tool (Kaplan-Meier plotter).

### Analysis of immune cell infiltration

The relative abundance of 22 immune cell types in the LUAD tumor microenvironment was estimated using the CIBERSORT algorithm. Samples with a CIBERSORT deconvolution p-value<0.05 were retained for subsequent analysis to ensure reliability. The correlation between CLK1 expression and immune cell infiltration levels was evaluated using the Gene Set Cancer Analysis (GSCA) database (Liu et al., 2023). The impact of CLK1 copy number variation on immune infiltration abundance was also assessed. The correlation between CLK1 and CD4^+^ T cells infiltration was further examined using the TIMER database (Cui et al., 2025). The predictive capability of CLK1 for immunotherapy response was comprehensively evaluated by plotting Receiver Operating Characteristic (ROC) curves via the immunotherapy prediction module. Finally, the infiltration abundance of immune cells was compared between CLK1 high-expression and low-expression groups using the Infiltration Estimation module.

### Single-cell RNA sequencing data analysis

The single-cell sequencing (scRNA-seq) data is sourced from GEO Dataset (GSE131907), which includes scRNA-seq of 58 lung adenocarcinoma samples from 44 patients. In this study, we used data from normal lungs (11 scRNA seq, labeled Normal) and lung adenocarcinoma tumors (11 scRNA seq, labeled Tumor). The scRNA-seq data analysis was performed as follows. First, the raw UMI count matrix and the cell annotation information provided by the dataset were loaded using the Seurat package in R. Cells derived from normal lung tissues and lung adenocarcinoma tissues were selected based on sample identifiers. Data preprocessing and quality control (QC) were then carried out. For the raw count matrix of the GSE131907 dataset, a Seurat object was constructed using the CreateSeuratObject function with parameters set as min. cells = 3 (genes detected in at least 3 cells were retained) and min. features = 200 (cells with at least 200 detected genes were retained). For QC, the percentage of mitochondrial genes (prefixed with “MT-”) per cell was calculated using PercentageFeatureSet and stored as percent. mt. Low-quality cells were filtered out using the following criteria: nFeature_RNA>200, nFeature_RNA<6000, and percent. mt<20%. The filtered data were then normalized using the LogNormalize method (scale factor = 10000) and linearly scaled via the ScaleData function. For dimensionality reduction and clustering, principal component analysis (PCA) was first applied for linear dimensionality reduction. The top 30 principal components, determined by the elbow method, were used for subsequent non-linear reduction (UMAP) and unsupervised clustering. Clustering was performed using the FindNeighbors (based on the selected PCs) and FindClusters functions (Louvain algorithm). The stability of clusters across different resolutions was evaluated with the clustree package, and a resolution of 0.6 was selected for final cell clustering. The resulting clusters were annotated according to the original cell type labels provided by the dataset, which were defined based on known marker gene expression. Annotation consistency was visually verified via UMAP projection. Finally, based on the expression level of CLK1, each cell subpopulation was classified into either CLK1 positive (high expression) or CLK1 negative (low expression) groups.

### Drug sensitivity analysis

Drug sensitivity data were obtained from the Genomics of Drug Sensitivity in Cancer (GDSC) and the Cancer Therapeutics Response Portal (CTRP) databases. Compounds whose half-maximal inhibitory concentration (IC_50_) showed a significant correlation with CLK1 mRNA expression (Pearson correlation, p < 0.05) were identified. For the NCI-60 cell line panel, drug sensitivity data (measured by AUC) and CLK1 expression were retrieved from the CellMiner database (https://discover.nci.nih.gov/cellminer/). Pearson correlation analysis was performed to identify drugs for which sensitivity was significantly associated with CLK1 expression levels.

### Statistical analysis

Data from *in vitro* experiments are presented as mean ± Standard Error of the Mean (SEM). For bioinformatics and clinical data analyses, specific statistical methods were applied as follows: Univariate analyses of clinical features employed the Chi-square test for categorical variables and the Wilcoxon rank-sum test for continuous variables. Survival analysis was performed using the Kaplan-Meier method, with differences between groups assessed by the log-rank test. To identify independent prognostic factors, multivariate analysis was conducted using the Cox proportional hazards regression model; results are presented as hazard ratios (HR) with 95% confidence intervals (CI) in Table 1 and corresponding forest plots. Correlation analyses between continuous variables were evaluated using Pearson’s correlation coefficient. For *in vitro* experiments comparing two groups, the unpaired two-tailed Student’s t-test was used; comparisons among multiple groups were performed using one-way ANOVA. A two-sided p-value<0.05 (or an adjusted p-value/FDR where specified) was considered statistically significant for all tests. All statistical and bioinformatics analyses were performed using R software (version 4.2.2).

**TABLE 1:** Correlation between CLK1 expression and clinical features.

Characteristics	CLK1 high (n = 318)	CLK1 low (N = 137)	P-value	Effect size/Correlation	Odds ratio (95% CI)
Age (year)					
<65	141 (44.3%)	58 (42.3%)	0.350	φ = 0.068	-
≥65	168 (52.8%)	78 (56.9%)	​	​	​
Unknown	9 (2.8%)	1 (0.7%)	​	​	​
Gender					
Female	173 (54.4%)	78 (56.9%)	0.647	φ = 0.022	1.14 (0.76–1.70)
Male	145 (45.6%)	59 (43.1%)	​	​	​
TNM stage					
I	184 (57.9%)	67 (48.9%)	0.163	-	-
II	74 (23.3%)	32 (23.4%)	​	​	​
III	45 (14.2%)	29 (21.2%)	​	​	​
IV	15 (4.7%)	9 (6.6%)	​	​	​
Residual tumor					
R0	222 (69.8%)	88 (64.2%)	1*	-	0.98 (0.33–2.94)
R1-R2	11 (3.5%)	5 (3.6%)	​	​	​
Unknown	85 (26.7%)	44 (32.1%)	​	​	​
Living status					
Alive	216 (67.9%)	68 (49.6%)	<0.001	φ = 0.178	2.14 (1.43–3.22)
Dead	102 (32.1%)	69 (50.4%)	​	​	​
Disease status					
NO	138 (43.4%)	53 (38.7%)	0.008*	φ = 0.161	0.51 (0.27–0.96)
Unknown	112 (35.2%)	70 (51.1%)	​	​	​
YES	68 (21.4%)	14 (10.2%)	​	​	​

Data are presented as n (%). P-values were calculated using chi-square test or Fisher’s exact test (*). OR, odds ratio; CI, confidence interval. OR>1 indicates higher risk in CLK1 low group; OR<1 indicates higher risk in CLK1 High group. Significant differences (P < 0.05) are highlighted.

## Results

### CLK1 is downregulated across multiple cancer types and holds prognostic value in LUAD

**FIGURE 1:**
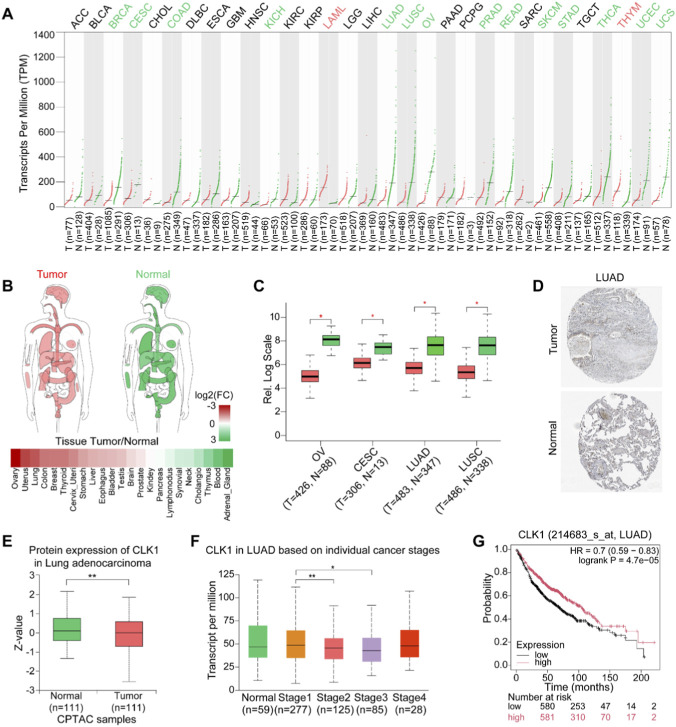
CLK1 is downregulated in NSCLC and correlates with favorable prognosis. **(A)** Pan-cancer analysis of CLK1 mRNA expression between tumor (T) and normal (N) tissues from the GEPIA database (http://gepia2.cancer-pku.cn/#index). ANOVA, |Log2FC| Cutoff = 1, q-value Cutoff = 0.01. **(B)** Differences in CLK1 mRNA expression between tumor and adjacent normal tissues across various cancer types. Data from GEPIA database. **(C)** Significant downregulation of CLK1 mRNA expression in OV, CESC, LUAD, and LUSC versus paired normal tissues. Data from GEPIA database. **(D)** Representative immunohistochemistry images of CLK1 protein expression in LUAD and normal lung tissues from LUAD patients in the Human Protein Atlas (HPA) database. **(E,F)** Protein expression of CLK1 in tumor versus normal tissues from LUAD patients **(E)** and its association with tumor progression **(F)** using CPTAC data. Data from https://ualcan.path.uab.edu/. **(G)** Kaplan-Meier overall survival curves for LUAD patients stratified by median CLK1 expression (KM plotter). High CLK1 expression is associated with significantly better survival (HR = 0.70, 95% CI: 0.59–0.83, log-rank P = 4.7e-5). *, p < 0.05; **, p < 0.01.

To investigate the role of CLK1 in carcinogenesis, we first analyzed its pan-cancer expression profile using the GEPIA database. CLK1 was significantly downregulated in tumor tissues relative to matched normal tissues in 14 cancer types—including CESC, LUAD, LUSC, and OV—compared to normal counterparts (Figures 1A–C). Given that lung cancer remains one of the most common and lethal malignancies (Bray et al., 2024), we focused subsequent investigations on this cancer type. This downregulation of CLK1 in tumor tissues was confirmed at the protein level in NSCLC using immunohistochemistry and proteomic data (Figures 1D,E). In LUAD, lower CLK1 expression was associated with more advanced tumor stage (stage2 vs. stage1, p = 0.008; stage3 vs. stage1, p = 0.03; Figure 1F). To evaluate the clinical relevance of CLK1 downregulation, we conducted survival analysis. Kaplan-Meier analysis demonstrated that high CLK1 expression was associated with significantly prolonged overall survival (log-rank p = 4.7 × 10^−5^) and a 30% reduction in the risk of death in LUAD patients (Hazard Ratio [HR] = 0.70, 95% Confidence Interval [CI]: 0.59–0.83; Figure 1G). These results collectively indicate that CLK1 is downregulated in multiple cancers, including LUAD, and carries significant prognostic value in this setting.

**FIGURE 2:**
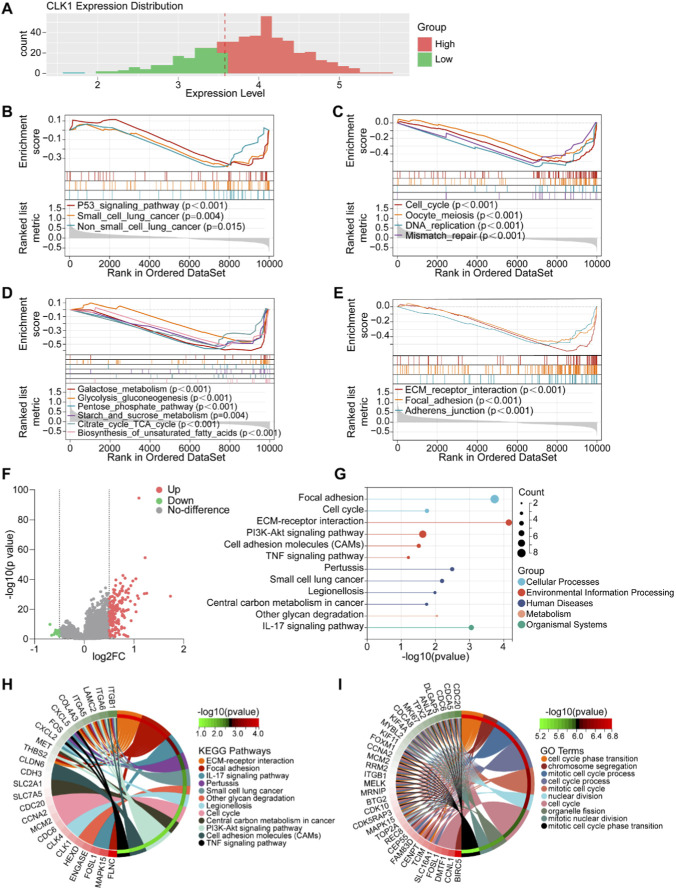
Functional enrichment analysis of CLK1-associated pathways in LUAD. **(A)** Grouping based on the median CLK1 expression value. N = 455, CLK1-high group (318) and CLK1-low group (137). **(B–E)** Gene Set Enrichment Analysis (GSEA) results showing pathways significantly suppressed in the CLK1-high group, including p53 signaling **(B)**, cell cycle processes **(C)**, metabolic pathways **(D)**, and cell adhesion **(E)**. **(F)** Volcano plot displaying the distribution of DEGs between CLK1-high and CLK1-low groups (FC ≥ 1.5, p < 0.05). **(G–I)** Kyoto Encyclopedia of Genes and Genomes (KEGG) **(G,H)** and Gene Ontology (GO) **(I)** enrichment analysis of the identified DEGs.

To determine if CLK1 is an independent prognostic biomarker, we analyzed data from 455 LUAD patients from the TCGA database. Patients were stratified into high- and low-CLK1 expression groups using the median expression value as the cutoff, which placed 318 patients in the high-expression group and 137 in the low-expression group (Figure 2A; Table 1). The associations between CLK1 expression and clinical features were assessed using chi-square or Fisher’s exact tests. CLK1 expression was significantly associated with living status (P < 0.001) and disease status (P = 0.008), but not with age, gender, TNM stage, or residual tumor status (all P > 0.05). Specifically, low CLK1 expression was associated with higher mortality (OR = 2.14, 95% CI: 1.43–3.22), while high CLK1 expression was associated with a higher rate of disease progression/recurrence (OR = 0.51, 95% CI: 0.27–0.96). These results suggest that CLK1 may serve as a prognostic biomarker with distinct implications for survival and disease progression in LUAD.

### Functional enrichment analysis of CLK1-associated signaling pathways in LUAD

To investigate the biological functions of CLK1, we performed gene set enrichment analysis (GSEA) comparing LUAD patients with high (n = 318) vs. low CLK1 (n = 137) expression. Elevated CLK1 expression suppressed the P53 signaling pathway, which correlated with slower tumor progression in NSCLC patients relative to those with low CLK1 expression was associated with significant suppression of oncogenic pathways, including the cell cycle (e.g., P53 signaling pathway, p < 0.001, DNA replication, p < 0.001; mismatch repair, p < 0.001) (Figures 2B,C). In cellular metabolism, high CLK1 expression strongly inhibited multiple metabolic pathways vital for tumor proliferation, including galactose metabolism (p < 0.001), glycolysis/gluconeogenesis (p < 0.001), the pentose phosphate pathway (p < 0.001), starch and sucrose metabolism (p = 0.004), the citrate cycle (TCA cycle) (p < 0.001), and the biosynthesis of unsaturated fatty acids (p < 0.001) (Figure 2D). Numerous studies have shown that changes in key metabolic pathways can directly affect cell proliferation, differentiation, aging, and even carcinogenesis (Li et al., 2023; Ma et al., 2021; Ma and Xu, 2024; Dai et al., 2025). Moreover, high CLK1 expression substantially affected pathways related to cellular connection and adhesion, such as ECM-receptor interaction, focal adhesion, and adherens junction (Figure 2E). A volcano plot constructed from the DEGs revealed that most genes were upregulated (Figure 2F). Pathway enrichment analysis of these significant DEGs further highlighted involvement in proliferation and immune-related processes (Figures 2G–I). These findings imply that CLK1 may may inhibit tumor progression in LUAD patient by constraining cell proliferation and metabolic reprogramming.

### CLK1 expression correlates with an immunologically active tumor immune microenvironment

**FIGURE 3:**
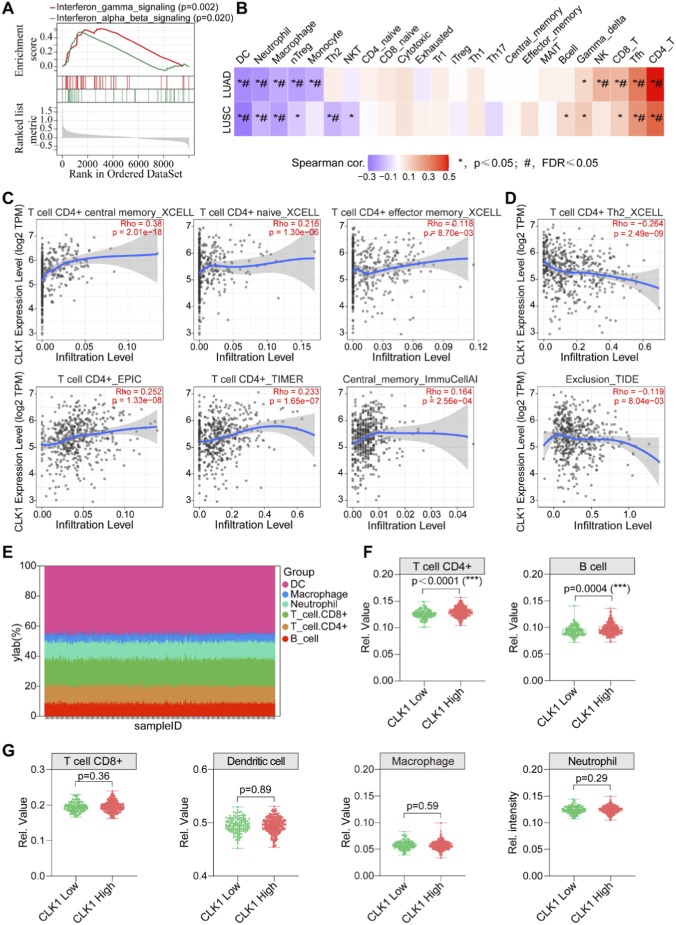
CLK1 expression is associated with an immunologically active tumor microenvironment. **(A)** GSEA show significant enrichment of type I and II interferon response pathways in the CLK1-high group. **(B)** Correlation heatmap between CLK1 expression and the infiltration abundance of 22 immune cell types was performed using the immune module of the GSCA database. **(C,D)** Detailed correlation analysis between CLK1 expression and specific CD4^+^ T cell subtypes using the TIMER database. **(E)** Composition of major immune cell types in the LUAD cohort (n = 455) estimated by the Estimation module of TIMER. **(F,G)** Comparative analysis of immune cell infiltration between CLK1-high and CLK1-low groups. The abundances of CD4^+^ T cells, B cells, CD8^+^ T cells, dendritic cells, macrophages, and neutrophils were evaluated. Infiltration levels of CD4^+^ T cells and B cells were significantly higher in the CLK1-high group. ***, P < 0.001.

Given the link between metabolism and immunity, we investigated CLK1’s role in the tumor immune landscape. GSEA indicated significant enrichment of type I interferon (IFN-α/β) (p = 0.02) and type II interferon (IFN-γ) (p = 0.002) response pathways in the high-CLK1 group (NES>1.5, FDR<0.05), suggesting immune activation (Figure 3A). To investigate the relationship between CLK1 and tumor immunity, we first assessed the correlation between CLK1 expression and immune cell abundance in LUAD using the Gene Set Cancer Analysis (GSVA) method developed by Liu et al. (2023). The results demonstrated that high CLK1 expression positively correlated with anti-tumor immune cell such as particularly CD4^+^ T cells (LUAD, r = 0.49, FDR = 2.87e-34, p = 3.27e-36, LUSC, r = 0.42, FDR = 3.92e-24, p = 2.99e-25), and negatively correlated with immunosuppressive subsets like, neutrophils (LUAD, r = −0.20, FDR = 1.92e-5, p = 2.18e-6, LUSC, r = −0.13, FDR = 0.009, p = 0.002) and M2 macrophages (LUAD, r = −0.17, FDR = 8.4e-5, p = 2.48e-5, LUSC, r = −0.13, FDR = 0.005, p = 0.003) (Figure 3B). These findings suggest that high CLK1 expression may promote tumor immunity.

Tumor Immune Estimation Resource (TIMER) is a systematic tool for analyzing the correlation between individual genes and immune infiltration (Cui et al., 2025). Given the significant positive correlation between CLK1 expression and CD4^+^ T cells, we further employed TIMER 3 to examine the association between CLK1 and various CD4^+^ T cell subsets. The results indicated that CD4^+^ T cell subsets confirmed CLK1 strong positive correlations with effector and memory populations, including T cell CD4^+^ central memory (XCELL) (r = 0.38, p = 2.01e-18), T cell CD4^+^ naive (XCELL) (r = 0.216, p = 1.30e-06), T cell CD4^+^ effector memory (XCELL) (r = 0.118, p = 8.70e-03), T cell CD4^+^ (EPIC) (r = 0.252, p = 1.33e-08), T cell CD4^+^ (TIMER) (r = 0.233, p = 1.65e-07), and central memory (immuCellAI) (r = 0.164, p = 2.56e-04) (Figure 3C). Conversely, CLK1 expression was negatively correlated with immunosuppressive subsets such as T cell CD4^+^ Th2 (XCELL) (r = −0.264, p = 2.49e-0) and Exclusion (TIDE) (r = −0.119, p = 8.04e-03) (Figure 3D). These results confirm a strong positive correlation between CLK1 expression and CD4^+^ T cell immune infiltration.

To further investigate the mechanism of CLK1 in tumor immunity, we compared immune cell infiltration levels between CLK1-high and CLK1-low LUAD patients (n = 455) using the Infiltration Estimation module of TIMER3. The proportions of immune infiltration by DCs, macrophages, neutrophils, CD8^+^ T cells, CD4^+^ T cells, and B cells were visualized (Figure 3E). Subsequent analysis revealed that CD4^+^ T cell infiltration was significantly elevated in the CLK1 high group (p < 0.0001, Figure 3F). A modest increase in B cell infiltration was also observed, whereas the abundances of dendritic cells, macrophages, neutrophils, and CD8^+^ T cells did not differ significantly between groups (Figures 3F,G). These findings further support the notion that CLK1 expression is strongly positively correlated with CD4^+^ T cell infiltration and that high CLK1 expression likely activates tumor immunity primarily by enhancing CD4^+^ T cell involvement.

### Tumor mutational burden (TMB) analysis and the effect of CLK1 on tumor immunity

**FIGURE 4:**
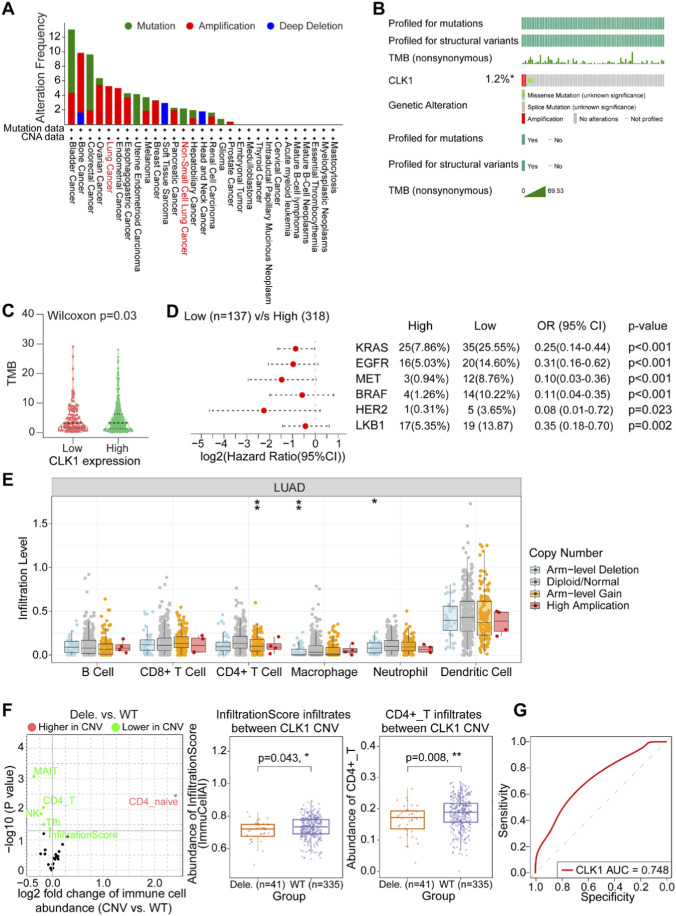
Association of CLK1 with tumor mutational burden, genomic alterations, and immunotherapy response. **(A,B)** Overview of CLK1 genomic alterations across cancers and in LUAD. Data from cBioPortal (https://www.cbioportal.org/) **(C)** Tumor Mutational Burden (TMB) is significantly higher in the CLK1-high group compared to the CLK1-low group. **(D)** Forest plot analysis of mutation frequencies of several cancer-driving genes in CLK1 low-expression and high-expression groups. **(E)** Impact of CLK1 copy-number variation (CNV) and the infiltration levels of specific immune cells. Data from TIMER3 (https://compbio.cn/timer3/) **(F)** Analysis of the effect of CLK1 copy-number loss on overall immune infiltration scores and key anti-tumor immune cells Data from GSCA database (https://guolab.wchscu.cn/GSCA/#/) **(G)** Receiver operating characteristic (ROC) curve illustrating the predictive value of CLK1 expression for immune infiltration in status (AUC = 0.748). *, P < 0.05; **, P < 0.01; ***, P < 0.001.

Given that a high correlates with increased neoantigen generation and enhanced response to immune checkpoint inhibitor (ICI) therapy, we evaluated whether CLK1 expression is associated with TMB in the LUAD cohort. To evaluate the association between CLK1 and TMB, we first analyzed CLK1 expression and its genomic alterations in LUAD using cBioPortal (https://www.cbioportal.org/). The results showed a relatively high mutation rate in patients with lung cancer and non-small cell lung cancer (Figure 4A). The mutation frequency of CLK1 was 1.2% among 729 LUAD cases (Figure 4B). Notably, the median nonsynonymous mutation counts in the TMB of these 729 LUAD patients was as high as 69.53, suggesting strong intrinsic immunogenicity and favorable responsiveness to immunotherapy in this cohort (Figure 4B). Compared TMB between groups and revealed a significantly higher burden in CLK1-high group (Wilcoxon p = 0.03, Figure 4C), indicating that elevated CLK1 expression is associated with a tumor microenvironment more likely response to immunotherapy.

We next investigated the relationship between CLK1 expression and key driver gene mutations in LUAD. Analysis revealed that mutations in several oncogenic drivers were significantly enriched in the CLK1 low-expression group. Specifically, KRAS mutation was present in 25.55% (35/137) of the low-expression group compared to only 7.86% (25/318) in the high-expression group (OR = 0.25, 95% CI: 0.14–0.44, *P* < 0.001). Similarly, we observed a significantly higher mutation frequency in the CLK1 low-expression group for EGFR (14.60% vs. 5.03%, OR = 0.31, 95% CI: 0.16–0.62, *P* < 0.001), MET (8.76% vs. 0.94%, OR = 0.10, 95% CI: 0.03–0.36, *P* < 0.001), BRAF (10.22% vs. 1.26%, OR = 0.11, 95% CI: 0.04–0.35, *P* < 0.001), HER2 (3.65% vs. 0.31%, OR = 0.08, 95% CI: 0.01–0.72, *P* = 0.023), and LKB1 (13.87% vs. 5.35%, OR = 0.35, 95% CI: 0.18–0.70, *P* = 0.0021). Forest plot visualization of these data consolidated the strong negative association between high CLK1 expression and the mutational burden of these key drivers (Figure 4D). This consistent pattern across multiple oncogenic pathways strongly suggests that CLK1 may function as a broad-spectrum tumor suppressor in LUAD.

Copy number alteration analysis further delineated the immunomodulatory role of CLK1. Arm-level gain of CLK1 was specifically associated with significantly enhanced infiltration of CD4^+^ T cells (Figure 4E). Conversely, CLK1 deletion correlated with a globally suppressed immune microenvironment, characterized by significantly reduced score for overall immune infiltration and key anti-tumor cell types including CD4^+^ T cells and natural killer (NK) cells (Figure 4F). To directly evaluate the translational potential of this association, we assessed the predictive capacity of CLK1 expression for immunotherapy response. Receiver operating characteristic (ROC) curve analysis yielded an area under the curve (AUC) of 0.748 for CLK1, indicating a robust predictive value for CLK1 in distinguishing likely responders to immune checkpoint blockade (Figure 4G).

### Single-cell RNA sequencing reveals CLK1-associated immune landscape in LUAD.

**FIGURE 5:**
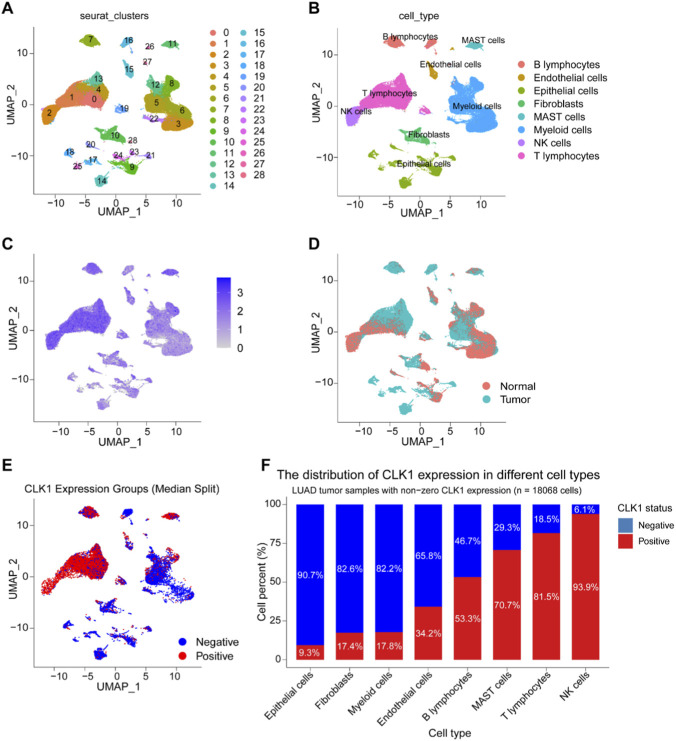
Single-cell RNA sequencing reveals the immune landscape associated with CLK1 in LUAD. **(A)** UMAP plot showed the total of 29 clusters were identified from the scRNA-seq dataset of 11 LUAD samples. 1–29 corresponding to different colors. **(B)** UMAP plot displaying the distribution of 8 distinct cell types. B lymphocytes, endothelial cells, epithelial cells, fibroblasts, mast cells, myeloid cells, NK cells and T lymphocytes. **(C)** The UMAP plot showed the heterogeneous expression of CLK1 in different cell subsets, the color gradient represents the expression level of CLK1. **(D)** The UMAP plots shows the difference in the position of cells from different sample sources (normal/tumor) in the reduced-dimensional space, reflecting the grouping characteristics of normal and tumor cells. **(E)** UMAP plot showed the distribution of CLK1 expression. The cells were divided into CLK1 high expression group (Positive, red) and low expression group (Negative, blue) by “Median expression”. **(F)** The proportion histogram of CLK1 expression in different cell types. The analysis object was “LUAD tumor sample cells with non-zero expression of CLK1 (n = 18068 cells)”; each column represents one cell type. The blue segment corresponds to “CLK1 Negative (low expression)”, and the red segment corresponds to “CLK1 Positive (high expression)”. The value on the right side of the column is the proportion of CLK1 Positive in each cell type (such as 9.3% in epithelial cells and 93.9% in NK cells).

To delineate the immune landscape associated with CLK1 at single-cell resolution, we analyzed scRNA-seq data from 11 patients with LUAD. Following quality control and normalization, cells were classified into 29 clusters (Figure 5A) and annotated into eight major lineages (Figure 5B). CLK1 expression exhibited marked heterogeneity across these cellular subsets (Figure 5C,D). Notably, among 18,068 tumor-derived cells with non-zero CLK1 expression, cells defined as CLK1-high were significantly enriched within immune cell clusters (Figure 5E). Quantification confirmed that CLK1-positive cells were highly enriched in NK cells and T cells (Figure 5F). This spatially resolved expression pattern strongly suggests that elevated CLK1 expression is closely linked to an immunologically “hot” tumor microenvironment in LUAD, providing single-cell-level evidence for its potential role in modulating anti-tumor immunity.

### Drug sensitivity analysis identifies compounds associated with CLK1 expression

**FIGURE 6:**
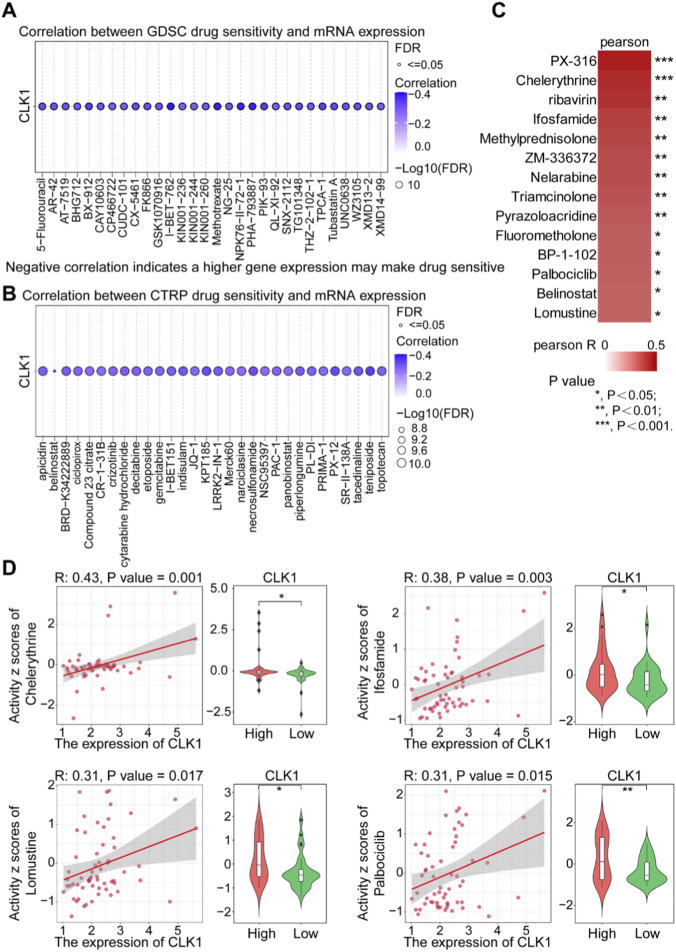
Drug sensitivity analysis identifies compounds associated with CLK1 expression. **(A,B)** Correlation analysis between CLK1 expression and drug sensitivity (IC_50_) from the GDSC **(A)** and CTRP **(B)** databases, highlighting broadly increased chemosensitivity in CLK1-high contexts. **(C)** Heatmap from the CellMiner (NCI-60) database showing 14 compounds with sensitivity positively correlated to CLK1 expression levels. **(D)** Validation of four specific compounds (chelerythrine, ifosfamide, lomustine, palbociclib) that exhibit significantly enhanced activity in CLK1-high models. *, P < 0.05; **, P < 0.01; ***, P < 0.001.

We next systematically evaluated the association between CLK1 expression and drug sensitivity. Initial screening using the GDSC and CTRP databases revealed a broad negative correlation between CLK1 expression and the half-maximal inhibitory concentration (IC_50_) of numerous compounds, suggesting that high CLK1 expression may confer general chemosensitivity (Figures 6A,B). To identify agents with positive correlation (i.e., higher efficacy in CLK1-high contexts), we interrogated the NCI-60 panel via the CellMiner database. This analysis pinpointed several candidates whose activity was positively linked to CLK1 mRNA levels (Figure 6C). Subsequent, validation confirmed that four compounds—chelerythrine, ifosfamide, lomustine, and palbociclib—exhibited significantly enhanced cytotoxic activity in cell line models with high CLK1 expression (Figure 6D). These results not only substantiate the role of CLK1 in modulating therapeutic response but also position it as a dual-purpose biomarker: predictive of both intrinsic chemosensitivity and specific vulnerability to targeted agents, thereby informing potential strategies for tailored therapy in NSCLC.

### CLK1 suppresses NSCLC cell proliferation *in vitro*

**FIGURE 7:**
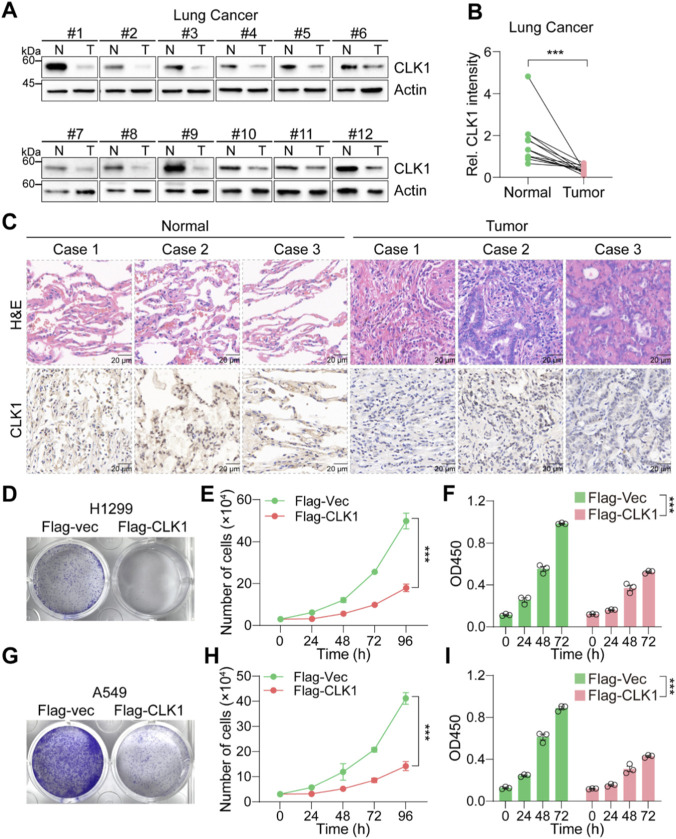
CLK1 suppresses NSCLC cell proliferation *in vitro*. **(A,B)** Expression of CLK1 in tumor tissues and adjacent non-tumor tissues from NSCLC patients was detected by immunoblotting. **(C)** Expression of CLK1 in tumor tissues and adjacent non-tumor tissues from NSCLC patients was detected by immunohistochemistry. **(D–F)** Overexpression of CLK1 in H1299 cells significantly inhibits colony formation **(D)**, reduces cell number over time **(E)**, and decreases cell viability **(F)** compared to control. **(G–I)** Consistent anti-proliferative effects of CLK1 overexpression in A549 cells across colony formation **(G)**, cell counting **(H)**, and CCK-8 assays **(I)**. ***, P < 0.001.

To functionally validate our bioinformatic findings, we confirmed that CLK1 protein was significantly downregulated in clinical NSCLC tumor samples compared to adjacent normal tissues (p < 0.01, Figures 7A–C). To further investigate the role of CLK1 in suppressing lung cancer cell proliferation, CLK1 was stably overexpressed in two NSCLC cell lines (A549 and H1299). Stable overexpression of CLK1 in A549 and H1299 NSCLC cell lines significantly inhibited cell proliferation, as demonstrated by colony formation assay, cell counting, and CCK-8 assay (all p < 0.001, Figures 7D–I). These results collectively establish CLK1 as a *bona fide* tumor suppressor in NSCLC.

## Discussion

This study identifies CLK1 as a novel tumor suppressor and positive immune regulator in NSCLC. We observed that CLK1 is consistently downregulated at the mRNA and protein levels in LUAD, and its low expression correlates with advanced disease stage and poor patient survival. Importantly, multivariate analysis confirmed CLK1 as an independent favorable prognostic factor. Functional analyses revealed that high CLK1 expression is linked to the suppression of cell cycle progression, key metabolic pathways, and oncogenic signaling. Most strikingly, CLK1 expression strongly correlated with an anti-tumor immune microenvironment, particularly enhanced infiltration of CD4^+^ T cells, a higher tumor mutational burden (TMB), and greater predicted sensitivity to immune checkpoint inhibition. Furthermore, we identified several chemotherapeutic agents, including clinically used ifosfamide and lomustine, with predicted higher efficacy in CLK1-high contexts. Finally, we experimentally validated that CLK1 is downregulated in clinical NSCLC samples and that its overexpression inhibits lung cancer cell proliferation *in vitro*.

CLK1, a Cdc2-like kinase, plays a significant role in alternative splicing (Haltenhof et al., 2020). It has been reported that CLK1 regulates influenza A virus mRNA splicing, and its inhibition prevents viral replication (Artarini et al., 2019). Our findings reveal a tumor-suppressive role for CLK1 in NSCLC, which contrasts with its reported oncogenic functions in other malignancies. In glioma and pancreatic ductal adenocarcinoma, high CLK1 expression promotes tumor growth, chemoresistance, and metastasis (Chen et al., 2021; Zhang et al., 2017). Additionally, CLK1 plays a critical role following drug treatment in peripheral T-cell lymphoma (PTCL), CLK1 is essential for the survival of residual tumor cells after treatment with cGAS inhibitors (Lu et al., 2024). This stark divergence underscores the profound tissue-specificity of CLK1 function. The mechanisms driving this context-dependency remain unknown but may involve cell-type-specific splicing substrates, differential interaction partners, or distinct upstream regulators. Our results highlight the critical importance of precisely defining the functional role of a target within a specific cancer type before considering therapeutic intervention.

A central and novel finding of our work is the robust association between CLK1 and an immunologically “hot” tumor microenvironment, specifically enriched for CD4^+^ T cells. While this study establishes a strong correlation, the molecular mechanism by which CLK1 might orchestrate immune cell infiltration warrants further investigation. As a kinase regulating alternative splicing (Artarini et al., 2019), CLK1 is poised to modulate the expression of immunomodulatory genes. We hypothesize that in NSCLC, CLK1 may influence the splicing or expression of key factors such as chemokines (e.g., CXCL9, CXCL10), cytokine receptors, or antigen presentation machinery, thereby shaping the immune landscape. Future studies employing splicing arrays or RNA-seq upon CLK1 modulation in tumor cells, followed by co-culture experiments with immune cells, are needed to identify the specific immunoregulatory pathways involved. This mechanistic insight could reveal new avenues for combining CLK1 modulation with immunotherapy.

Additionally, the link between high CLK1 expression, elevated TMB, and better predicted response to immunotherapy is clinically significant. TMB-high status generates neoantigens that facilitate immune recognition (Samstein et al., 2019). Our data suggest that CLK1-high tumors may be more immunogenic and thus more susceptible to immune attack. This aligns with the observed enrichment of interferon-signaling pathways and effector immune cells. Moreover, since the copy number variation of proto-oncogenes or tumor suppressor genes in tumor cells is associated with immune infiltration in tumor tissue (Dai and Liu, 2021). The association between CLK1 copy number loss and a suppressed immune microenvironment reinforces its role as a positive immune regulator. These insights position CLK1 as a potential dual biomarker: for prognosis and for predicting a favorable immune context.

The GDSC and the CTRP databases serve as novel resources for identifying therapeutic biomarkers in cancer cells. They integrate large-scale genomic datasets with drug sensitivity information, thereby enabling the prediction of drug sensitivity toward target genes and providing a theoretical basis for preclinical cancer research (Cortes-Ciriano et al., 2016; Yang et al., 2013; Iorio et al., 2016). Our drug sensitivity analysis extends the clinical relevance of CLK1 beyond prognosis. The identification of drugs like ifosfamide and lomustine, which are already used in NSCLC regimens, as having predicted higher activity in CLK1-high tumors is particularly compelling (Fossella et al., 2000; Manegold et al., 1992; Bozcuk and Artac, 2024; Seliger et al., 2022). This suggests that CLK1 expression could serve as a companion biomarker to stratify patients for these chemotherapies, potentially improving response rates. While palbociclib and chelerythrine are less established in NSCLC, they represent interesting candidates for future investigation (Wang et al., 2021; Zhou and Hu, 2025). These findings lay a theoretical foundation for exploring CLK1 as a tool for personalized therapy selection.

Several limitations of this study must be acknowledged. First, while we validated CLK1’s anti-proliferative effect *in vitro*, its tumor-suppressive and immunomodulatory roles require confirmation in vivo models. Second, the clinical correlations rely heavily on bioinformatic analyses of public datasets like TCGA, which may have inherent selection biases. Our in-house clinical sample size for protein validation was also limited. Third, the mechanistic link between CLK1 and CD4^+^ T cell infiltration remains correlative; direct experimental evidence is needed. Finally, the predictive value of CLK1 for drug sensitivity, though promising, is derived from computational models and requires rigorous preclinical and clinical validation.

In conclusion, we provide comprehensive evidence that CLK1 acts as a tumor suppressor and immune promoter in NSCLC. It correlates with favorable prognosis, an activated immune microenvironment, and enhanced sensitivity to specific chemotherapeutic agents. These multifaceted roles make CLK1 a compelling biomarker worthy of further investigation. Future work should focus on elucidating the molecular mechanisms underlying its immune-regulatory functions and validating its utility in guiding combination therapies in NSCLC.

## Conclusion

Our study demonstrates that CLK1 functions as a tumor suppressor in NSCLC, where it is frequently downregulated. Its overexpression directly inhibits cancer cell proliferation *in vitro*. Through comprehensive bioinformatics analysis, we further establish a novel and significant role for CLK1 in shaping the tumor immune microenvironment. A key and unique finding is its strong association with enhanced anti-tumor immunity, particularly the infiltration of CD4^+^ T cells, coupled with a higher tumor mutational burden. These multifaceted roles position CLK1 not only as a robust independent prognostic biomarker but also as a potential predictor of immunotherapy response and chemotherapy sensitivity.

To underscore the novelty and translational potential of our work, we highlight two forward-looking directions: First, the tissue-specific tumor-suppressive function of CLK1 in NSCLC, which contrasts with its oncogenic role in other cancers, necessitates further mechanistic studies to unravel the underlying context-dependent pathways. Second, the compelling link between CLK1 and an immunogenic tumor microenvironment warrants validation *in vivo* models and clinical cohorts. Ultimately, targeting CLK1 or leveraging its expression as a companion diagnostic may open new avenues for combination therapies, integrating immunotherapy or specific chemotherapeutic agents (e.g., ifosfamide, lomustine) to improve outcomes for NSCLC patients.

## Data Availability

The datasets presented in this study can be found in online repositories. The names of the repository/repositories and accession number(s) can be found in the article/supplementary material.
